# Supplementary material for: Enhanced echocardiographic assessment of intracardiac flow in congenital heart disease
Source: PLoS One. 2024 Mar 18;19(3):e0300709. doi: 10.1371/journal.pone.0300709 (PMC10947680; doi:10.1371/journal.pone.0300709)
Supplement: S1 Table — A table of reported means, standard deviations, and p-values for the flow measurement quantities obtained for each condition across each imaging modality. (DOCX) [file pone.0300709.s002.docx]

## S1 Table

Table 1: DoVeR and 4D Flow measurements obtained from analysis

| DoVeR |  |  |  |  |
| --- | --- | --- | --- | --- |
| Condition | RTOF (n=10) | RVD (n=3) | Normal (n=7) | *p* |
| VS_Maximum_ , s^-1^ | $22.9\pm11.3$ | $24.8\pm4.6$ | $17.2\pm11.5$ | $0.486$ |
| VS_Average_ , s^-1^ | $12.8\pm6.3$ | $13.5\pm5.1$ | $9.4\pm7.2$ | $0.514$ |
| VEL_Maximum_ , J/m^3^ | $3.9\pm2.8$ | $6.4\pm2.6$ | $5.6\pm3.9$ | $0.387$ |
| VEL_Average_ , J/m^3^ | $1.6\pm1.1$ | $2.9\pm1.8$ | $2.5\pm2.2$ | $0.410$ |
| KET_Maximum_ , W/m^3^ | $21.2\pm10.2$ | $33.5\pm12.4$ | $14.6\pm11.1$ | $\boldsymbol{0.065}$ |
| KET_Average_ , W/m^3^ | $6.9\pm3.0$ | $12.0\pm7.4$ | $5.8\pm5.2$ | $0.166$ |
| $\Delta P$_Suction_ , mmHg | $0.56\pm0.30$ | $0.56\pm0.40$ | $0.29\pm0.10$ | $0.143$ |
| $\Delta P$_Recovery_ , mmHg | $0.56\pm0.22$ | $0.78\pm0.43$ | $0.58\pm0.30$ | $0.512$ |
| 4D Flow MRI |  |  |  |  |
| Condition | RTOF (n=10) | RVD (n=3) | Normal (n=7) | *p* |
| VS_Maximum_ , s^-1^ | $24.3\pm7.4$ | $34.3\pm5.0$ | $27.7\pm16.7$ | $0.423$ |
| VS_Average_ , s^-1^ | $15.1\pm3.1$ | $22.4\pm7.3$ | $17.7\pm13.5$ | $0.446$ |
| VEL_Maximum_ , J/m^3^ | $6.4\pm3.5$ | $13.7\pm3.0$ | $14.5\pm24.0$ | $0.500$ |
| VEL_Average_ , J/m^3^ | $3.5\pm1.6$ | $7.0\pm4.2$ | $9.8\pm18.7$ | $0.537$ |
| KET_Maximum_ , W/m^3^ | $28.4\pm17.7$ | $85.0\pm37.2$ | $26.0\pm12.0$ | $\boldsymbol{<0.001}$ |
| KET_Average_ , W/m^3^ | $10.6\pm4.5$ | $32.0\pm11.5$ | $11.5\pm7.6$ | $\boldsymbol{<0.001}$ |
| $\Delta P$_Suction_ , mmHg | $0.43\pm0.18$ | $0.48\pm0.13$ | $0.20\pm0.11$ | $\boldsymbol{0.014}$ |
| $\Delta P$_Recovery_ , mmHg | $0.54\pm0.25$ | $0.93\pm0.22$ | $0.46\pm0.18$ | $\boldsymbol{0.021}$ |
