# Supplementary material for: Enhanced echocardiographic assessment of intracardiac flow in congenital heart disease
Source: PLoS One. 2024 Mar 18;19(3):e0300709. doi: 10.1371/journal.pone.0300709 (PMC10947680; doi:10.1371/journal.pone.0300709)
Supplement: S1 Text — A summarizing table of all abbreviations not commonly used in practice. (DOCX) [file pone.0300709.s003.docx]

## S1 File: Abbreviations

A4C = Apical 4 Chamber

CHD = Congenital heart defect

CMR = Cardiac magnetic resonance

DoVeR = Doppler Velocity Reconstruction

KET = Total kinetic energy

MRI = Magnetic resonance imaging

RV = Right ventricle

rTOF = repaired Tetralogy of Fallot

RVD = dilated right ventricle

VS = Vortex strength

VEL = Viscous energy loss

VFM = Vector flow mapping

venc = Velocity encoding

$\Delta P$ = Relative pressure difference
