# Supplementary material for: Enhanced echocardiographic assessment of intracardiac flow in congenital heart disease
Source: PLoS One. 2024 Mar 18;19(3):e0300709. doi: 10.1371/journal.pone.0300709 (PMC10947680; doi:10.1371/journal.pone.0300709)
Supplement: Attachment — Submitted filename: Response for Reviewers.docx [file pone.0300709.s004.docx]

## Overview and response structure

We thank the reviewers for their valuable input throughout the review process. We have addressed their revisions to clarify the presented work further. Each point is addressed with a response and the corresponding excerpt from the revised final manuscript. Our response is provided in red Arial, and the text excerpt is in blue Arial.

## Response to reviewers

**Reviewer #1:**

1. Were the echo and CMR imaging all done at one institution, or were there patients from both centers?

All echo and CMR was collected from Children’s National Hospital. We have added the following text entry to clarify this within the text.

All studies were performed within the Children’s National Hospital system.

2. Inclusion criteria are subjects with “diagnostic-level echo visualization of the RV,” then there are patients later excluded for poor color Doppler alignment. Can you expand on what is needed for color Doppler alignment for adequate analysis with DoVeR? In general, how challenging is it to obtain adequate echo imaging of the RV for DoVeR. I can see this being especially difficult in the adult congenital population where it also may be most useful.

We acknowledge that this statement is ambiguous and needs further clarification. We considered *poor color Doppler alignment* as a scan that does not capture the A4C plane with a clear view of the apex and tricuspid valve. We have added the following text entry to clarify this within the text.

Appropriate echo studies had color Doppler acquisition box over a majority of the RV chamber (particularly with an A4C alignment that adequately resolves both the tricuspid inflow apex), adequate temporal resolution (at least 10 frames per beat), adequate visualization of the RV free wall/septum, and appropriate color scaling to minimize aliasing of the flow.

The text excerpt above sets out the four criteria we used to select sufficient RV-aligned color Doppler scans from echocardiogram acquisitions retrospectively. Retrospective studies pose difficulties when used to study flow with color Doppler reconstruction. In this as in our previous studies, when collaborating with medical schools and research universities that specialize in treating patients with congenital heart defects, we have found that it is possible to create a retrospective cohort consisting of 70% to 80% of all individuals considered. However, this percentage will likely decrease when moving to less specialized care centers.

3. What was the reason for MRI on the cohort of normal patients?

The MRIs for the cohort of normal patients obtained for a separate clinical indication (exclude cardiomyopathy, atrial shunts or partial anomalous pulmonary venous return) and found to have normal right ventricular size, function and pulmonary-to-systemic flow ratio (Qp:Qs) < 1.2:1. We have added the following text to provide this information in the text.

The control subjects were obtained for a separate clinical indication and were found to have normal RV size, function, and pulmonary-to-systemic flow ratio (Qp:Qs < 1.2:1).

4. The text states for Figure 1 (d) as energy loss, and (e) as kinetic energy, whereas the figure states the opposite. Please clarify.

The text in the paper incorrectly references the fields in Figure 1. Energy loss is (e) and kinetic energy is (d). We have corrected the text to reference each field correctly.

5. Please expand how the 4D flow measurements are projected onto 2D space? Is this for visualization similar to a “maximal intensity projection (MIP)” would be for an angiogram? Is this for visualization only, or for quantification as well? Do the 4D flow measurements account for all of 3D space?

The 4D flow measurements were projected onto the 2D plane first by isolating the intracardiac flow from a 3D segmentation, then the 4D flow data with 2D plane parallel to the A4C view was isolated for extraction. Projection onto the 2D plane is done for visualization only. All quantitative comparisons are done between volume-averaged measurements from the full 4D flow MRI fields, which include all three velocity components. In order to clarify further in the text, we have amended the *Results* section for the *Similarity of DoVeR and 4D flow MRI flow dynamics*.

To enable visual and qualitative comparison with DoVeR, the 4D flow MRI measurements are projected from the volume onto the A4C plane. To project the 4D flow MRI measurements, the intracardiac flow was first isolated from a 3D segmentation, and then velocity measurements were interpolated linearly from the preserved 4D flow data onto a 2D plane parallel to the A4C view.

Finally, the 4D flow measurements account for all the 3D space. However, the flow within the RV was dynamically masked for each frame to preserve only velocities relevant to our region of interest for this study.

6. I understand that the small numbers of this study make this challenging, though can you discuss differences in the agreement of parameters between DoVeR and 4D flow in the different patient groups. Is there generally more agreement in normal patients vs rTOF patients as DoVeR is missing the RVOT which is captured in 4D flow?

We briefly touch on this point on page 13, starting at line 250, where we detail how more disturbed flow from pulmonary insufficiency may impact *R* and *L2-norms*, but that good agreement was still obtained. While acknowledging this limitation, we did not see any statistically significant difference between conditions for all quantities presented for both correlations and L2-norm differences. Thus, there was no better or worse agreement in normal patients vs rTOF patients when the RVOT is missed.

We have included a statement on testing the significance of *R* and *L2-norm differences* between subgroups in the Discussion section. The following text has been included in the manuscript.

While we explored differences in R and L2-norm between conditions, no statistically significant findings were observed, possibly due to the smaller subgroup sizes.

7. Would there be utility in repeating this analysis in other echocardiographic planes to capture RV outflow and pulmonary regurgitation?

Yes, there would be utility in future work that examines echo planes that capture the RV outflow. While correlation and L2-norm difference quantities are in agreement between 4D flow volume-averaged and 2D DoVeR area-averaged parameters did not show a difference, the magnitudes of the peaks for each quantity were not resolved, which is prevalent for energy loss. Analyzing fields with better alignments to capture the interaction of the filling and regurgitant jets is required to better resolve the magnitude.

**Reviewer #2:**

In this article, the authors aimed to compare the efficacy of echo DoVeR method with 4D flow MRI in quantifying intracardiac flow fields of the RV in some patients with congenital heart disease and controls. In general, the manuscript is well-structured and provides a clear context for your study. I have a few comments/suggestions:

Methods section:

1) Pg. 4, Line 74: The patient recruitment phase concluded in October 2020, extending beyond three years. Were there any additional patient enrolments during this period, and could you provide details regarding the study's timetable throughout? Additionally, given the retrospective nature of the study, it would be beneficial to specify the commencement date for the analysis of echo and MRI images. Clarifying this information will enhance the transparency of the study timeline.

The timeline of patient enrollment was dependent on date of IRB approval (October 2020) and when 4D flow imaging was incorporated into clinical practice at Children’s National (January 2018). Data collection proceeded through from October 2020 to June 2021. As this was a retrospective study, we did not subsequently enroll patients for the purposes of this validation study, although since June 2021 we have enrolled other cohorts for clinical research. We have added that 4D flow MRI was collected along with echo imaging to clarify the text. Furthermore, we have added the following text to further clarify the period of patient identification and data collection.

Patient data for use in this study was identified and collected from October 2020 to June 2021

2) Pg 4, Line 77: You enlisted three patients with right ventricular dysfunction (RVD) stemming from "atrial-level shunting." It would be helpful to clarify whether this atrial-level shunting exclusively involved atrial septal defects (ASD) in these patients or if other congenital heart diseases, such as partial anomalous pulmonary venous drainage, were also present. If the RVD was solely attributed to ASD, it might be more precise to indicate RVD in patients with ASD, avoiding the term "atrial-level shunting" to prevent confusion among readers. However, if other pathologies were involved in these three patients, please specify the nature of these conditions.

To clarify, RVD stands for RV dilation (not dysfunction), due to increased volume load from sinus venous defects with partial anomalous pulmonary venous return. This type of congenital heart disease contributes to the same physiological effect on the right ventricle as atrial septal defects - hence the term “atrial-level shunting”. We have further clarified this within the text in the *Study Population* subsection of *Methods*.

3) Pg 5, Line 83: “Echocardiogram Acquisition”. Provide a bit more detail on the echo imaging process. For example, mention the echo machine settings used, measures used to improve the temporal resolution and colour Doppler of your images, or any specific protocols followed during acquisition.

All echo imaging was performed following lab standard imaging protocols for RV assessment – on B-mode, there must be adequate visualization of the RV free wall motion. On B-mode and color Doppler, the sector width must be appropriately sized to cover the RV whilst also maintaining a frame rate was >20 Hz. The following text has been added to the text to provide this information.

Imaging was performed following lab standard imaging protocols for RV assessment. These include adequate visualization of the RV free wall motion for B-mode image acquisitions and sector width set to the appropriate size to cover the RV while also maintaining a frame rate > 20 Hz for both B-mode and color Doppler acquisitions.

Since these were retrospective acquisitions of clinical datasets, there were no specific protocols to maximize DoVeR capabilities. This was an intentional study design decision to assess the robustness of this methodology on retrospective clinical imaging. We have added a statement to the Discussion section to note this as a limitation and the need for further consideration in future studies.

Because this study used retrospective acquisitions of clinical data, no specific protocols to maximize DoVeR capabilities were utilized. This was an intentional aspect of our study design to assess the robustness of DoVeR for use with retrospective clinical imaging. Future studies will work to establish proper protocols to maximize DoVeR for use in RV flow analysis.

4) Pg 5, Line 85: “A routine A4C view of the RV”. I guess you mean A4C focused RV view to better visualize the free RV wall.

The reviewer is correct, albeit there are other requirements as well. While we did want to visualize the free wall of the RV better, we also wanted to have clear views of the apex and tricuspid valve. We have addressed this point in response to Reviewer #1, Comment #2 above.

5) Pg 6, Line 109: It's mentioned that a single DoVeR reconstruction takes approximately 7 minutes. While this information is useful, you may want to discuss the overall time required for the entire analysis process for each patient, including any preprocessing steps.

We acknowledge that this sentence reads as though the DoVeR reconstruction itself takes 7 minutes. However, from initialization to post-processing, with pre-processing and reconstruction included, the total time to run the comprehensive analysis on one color Doppler acquisition for an individual is 7 minutes. We have edited the original text entry to clarify this within the text.

A DoVeR reconstruction for a single dataset takes approximately 7 minutes to run, which covers the steps outlined above.

6) Pg 6, Line 114: “4D flow post-processing”. You mentioned that the velocity encoding was set between 2 m/s and 2.5 m/s during acquisition. Did you need VENC correction for any of your patients during processing?

As the focus of the intracardiac flow assessment was within the RV, we did not encounter any high velocity jets within that would require Venc correction that may be required in other clinical scenarios (e.g. assessment of mitral insufficiency/aortic insufficiency).

Results section:

1) Pg. 9, Line 161: What is meant be poor CMR alignment? Please clarify.

The clinical 4D flow datasets were acquired in a sagittal orientation thus the number of slices/acquisition time was dependent on the cardiac width. In some instances, the 4D flow datasets did not have enough slices to fully cover the RV apex (albeit still diagnostic quality as all great vessels/semilunar valves and atrioventricular valves were covered). This inadequate coverage of the RV apex precluded comparisons against echo imaging. We have edited the text to reflect this exclusion scenario.

For 6 cases (23%), either poor color Doppler acquisition settings or poor CMR alignment, where the 4D flow datasets did not have enough slices to cover the RV apex fully, prevented further analysis.

Discussion section:

Discussion of the study's limitations is crucial. Firstly, the limited size of the cohort, with only 13 patients compared to 7 controls, raises concerns. To ensure the robust validation of the echo DoVeR against 4D MRI, a larger sample size is recommended. Secondly, the utilization of single plane 2D echocardiography for reconstructing flow in the complex 3D geometry of the RV is another limitation. Consideration of future endeavours involving coloured 3D echocardiography of the RV, coupled with your semi-automated MATLAB algorithms, could enhance the study. This approach has the potential to reconstruct a 3D colour Doppler velocity, providing a more comprehensive three-dimensional dataset that may better align with data obtained from 4D MRI.

We acknowledge that a discussion on the limitations of the study design is lacking. We have added the following text to the Discussion section to address this and to clarify the text.

Our study has limitations that need acknowledgment. We had 26 subjects in our cohort, with varying numbers in each condition subgroup. Although our study aim is to compare DoVeR and 4D flow MRI agreement, differences in performance across conditions might exist. Additionally, 8 subjects (23% of the cohort) were excluded, potentially further weakening our measurements' significance. While we explored differences in R and L2-norm between conditions, no statistically significant findings were observed, possibly due to the smaller subgroup sizes. This could impact the power and significance of agreement between subgroups and underscores the need for a larger sample in future studies.

We also acknowledge that a discussion on the limitation of the DoVeR method is lacking. We have added the following text to the discussion section to address this and to clarify the text.

The DoVeR method similarly has a few limitations. Its current formulation is solely in 2D, potentially missing key aspects of highly 3D flows, like those in the RV, impacting the capture of relevant flow features. Our future focus includes expanding this method to 3D for reconstructing flows observed in 3D color Doppler recordings of the RV. DoVeR reconstruction can fail to provide a reliable reconstruction if the RV free wall is absent, improper tracking of the tricuspid annular plan occurs, or insufficient color signal is present during diastolic inflow. In these cases, either an inconsistent segmentation is rendered, which leads to an erroneous velocity field output, or the underlying flow physics cannot be properly satisfied. Addressing these limitations is an ongoing focus of our research efforts.

Table 1:

1) I believe that RV measurements (EDVi, ESVi, EF, and PR%) are all obtained from CMR not echocardiography. This should be stated.

Yes, we can confirm that all RV measurements were obtained from CMR. We have added the following text to indicate that CMR was used to obtain these measurements.

Reported RV measurements are collected from CMR imaging.

2) Is there a particular rationale behind not assessing the QP/QS ratio in patients with rTOF? Some of these patients may have residual shunts across the VSD patch. Even with the presence of severe PR, it's possible to quantify the QP/QS ratio in these individuals using CMR. Could you provide clarification on this matter?

In our original composition of the manuscript, we left this calculation out because it did not show separation from the control group, unlike the RVD. We have provided this quantity in Table 1 in the revised manuscript.

Figures:

1) The quality of the figures is suboptimal and requires enhancement.

Each figure has been run through PACE to enhance image quality.

**Reviewer #3:**

The authors compared a novel technique for estimating intracardiac flow dynamics from echocardiography against the more established but not widely available 4d flow MRI. The technique and the results are interesting. I have no major comments on the used methodology.

Minor comments:

1 - Line 116: used to segment…

The grammatical error has been addressed.

2 - Line 190: define acronym on first use (CFI)

This was a previously used acronym for *Color Flow Imaging*, a surrogate to color Doppler imaging, which has been removed. To address this in the text, *CFI* has been replaced with *color Doppler*.

3 - Figure 1: please indicate where the time series is being measured. Is it the mean time series of the whole RV area or taken from a specific point/region?

There were several strategies to collect the timeseries measurement based on the quantity of interest. Each strategy is as follows:

- Velocity Magnitude: The 97.5% percentile velocity across an area or volume near the tricuspid valve depending on modality used; this was not taken at a single point.
- Pressure difference: Difference between the minimum and maximum pressure location within the area or volume depending on modality.
- Vortex Strength: Regions of same-signed vorticity were integrated over the area or volume depending on modality, and summed together to obtain a measure of total vortex strength.
- Energy Loss and Kinetic energy: A quantity of interest was integrated over the area or volume depending on modality, resulting in a measure of the total quantity.

An explanation on how each of the timeseries quantities was measured was explained in the *Quantitative analysis* section of the *Methods*. However, the following text has been included for the inflow velocity measurement to clarify the text further.

The inflow velocity was measured from the tricuspid valve to 1 cm into the RV as the 97.5% quantile for each frame.

Furthermore, we have edited the text of Figure 1 caption to further clarify that each the timeseries measurements are volume averaged.

4 - Figure: likewise done with a healthy control in Figure 1, it would be interesting to show the time series from a representative CHD patient with low RV function. Or maybe add the time series to the slices shown in Figure 3.

This is an excellent suggestion. Figure 3 has been edited to include the timeseries measurements for each of the shown streamtrace fields as well, as shown below.

5 - Results section: consider moving any interpretations of the results to the discussion, e.g. line 227-228.

We have attempted to address any interpretations made to the results by moving them to the discussion.

6 - The values in Table S1 are of great interest. Consider moving these to the main manuscript body and cite the table in the text.

While we see the potential interest of the findings presented in Table S1, adding this information in the body of the manuscript may distract from the overall objective of the study. Additionally, the information reported in this table is based on a small cohort of patients; we believe more cases are needed to make the measurements in this table more meaningful. We also have reported similar information in a prior manuscript for the DoVeR based measurements,

Meyers, B., Nyce, J., Zhang, J., Frank, L. H., Balaras, E., Vlachos, P. P., & Loke, Y. H. (2023). Intracardiac Flow Analysis of the Right Ventricle in Pediatric Patients With Repaired Tetralogy of Fallot Using a Novel Color Doppler Velocity Reconstruction. *Journal of the American Society of Echocardiography*, *36*(6), 644-653.

We have referenced the table in the manuscript to direct interested readers to the supplemental.

Additional peak and average parameter statistics for all conditions are reported in supplemental material table S1. We direct interested readers here to further understand the differences between observed quantities in this study.

7 - Discuss the limitation of small study participants (especially after excluding 8 cases due to failed reconstruction). Also discuss factors of reconstruction failure as a limitation of the technique.

These points have been addressed in response to Reviewer #2 above.
